# Phenotypes of prediabetes and metabolic risk in Caucasian youths with overweight or obesity

**DOI:** 10.1007/s40618-022-01809-3

**Published:** 2022-05-17

**Authors:** P. Di Bonito, M. R. Licenziati, D. Corica, M. G. Wasniewska, A. Di Sessa, E. Miraglia del Giudice, A. Morandi, C. Maffeis, M. F. Faienza, E. Mozzillo, V. Calcaterra, F. Franco, G. Maltoni, G. Valerio

**Affiliations:** 1 Department of Internal Medicine, “S. Maria Delle Grazie” Hospital, Pozzuoli, Italy; 2 Obesity and Endocrine Disease Unit, Department of Neuroscience, Santobono-Pausilipon Children’s Hospital, Naples, Italy; 3 Department of Human Pathology in Adulthood and Childhood, University of Messina, Messina, Italy; 4 Department of Woman, Child and of General and Specialized Surgery, University of Campania “Luigi Vanvitelli”, Naples, Italy; 5 Department of Surgery, Dentistry, Pediatrics and Gynecology, Section of Pediatric Diabetes and Metabolism, University and Azienda Ospedaliera, Universitaria Integrata of Verona, Verona, Italy; 6 Department of Biomedical Sciences and Human Oncology, Pediatric Unit, University of Bari “Aldo Moro”, Bari, Italy; 7 Section of Pediatrics, Department of Translational Medical Science, Regional Center of Pediatric Diabetes, University of Naples “Federico II”, Naples, Italy; 8 Pediatric Department, “V. Buzzi” Children’s Hospital, Milan, Italy; 9 Department of Internal Medicine, University of Pavia, Pavia, Italy; 10 Pediatric Department, Azienda Sanitaria Universitaria del Friuli Centrale, Hospital of Udine, Udine, Italy; 11 Pediatric Unit, IRCCS Azienda Ospedaliero-Universitaria di Bologna, Bologna, Italy; 12 Department of Movement Sciences and Wellbeing, University of Naples “Parthenope”, via Medina 40, 80133 Naples, Italy

**Keywords:** Pediatric obesity, Prediabetes, Impaired fasting glucose, Impaired glucose tolerance, HbA1c, Cardiometabolic risk factors

## Abstract

**Purpose:**

To assess the prevalence of pre-diabetes phenotypes, i.e., impaired fasting glucose (IFG), impaired glucose tolerance (IGT), increased HbA1c (IA1c), and their association with metabolic profile and atherogenic lipid profile in youths with overweight/obesity (OW/OB).

**Methods:**

This cross-sectional study analyzed data of 1549 youths (5–18 years) with OW/OB followed in nine Italian centers between 2016 and 2020. Fasting and post-load measurements of glucose, insulin, and HbA1c were available. Insulin resistance (IR) was estimated by HOMA-IR and insulin sensitivity (IS) by reciprocal of fasting insulin. The atherogenic lipid profile was assessed by triglycerides-to-HDL ratio or cholesterol-to-HDL ratio. Insulinogenic index was available in 939 youths, in whom the disposition index was calculated.

**Results:**

The prevalence of overall pre-diabetes, IFG, IGT and IA1c was 27.6%, 10.2%, 8% and 16.3%, respectively. Analyzing each isolated phenotype, IGT exhibited two- to three-fold higher odds ratio of family history of diabetes, and worse metabolic and atherogenic lipid profile vs normoglycemic youths; IFG was associated only with IR, while IA1c showed a metabolic and atherogenic lipid profile intermediate between IGT and IFG.

**Conclusion:**

Prevalence of pre-diabetes was high and IA1c was the most prevalent phenotype in Italian youths with OW/OB. The IGT phenotype showed the worst metabolic and atherogenic lipid profile, followed by IA1c. More studies are needed to assess whether HbA1c may help improving the prediction of diabetes.

**Supplementary Information:**

The online version contains supplementary material available at 10.1007/s40618-022-01809-3.

## Introduction

Obesity (OB) affects millions of youths worldwide and imposes early in the life a heavy burden of cardiovascular and metabolic comorbidities, such as hypertension, dyslipidemia, liver steatosis and abnormal glucose regulation. In particular, it has been estimated that the prevalence of type 2 diabetes (T2DM) among adolescents will quadruple by 2050 [1]. Specifically, pre-diabetes represents an emerging clinical priority in the setting of pediatric obesity [2] since it has increased at an alarming rate among obese youths [3]. Furthermore, it may be associated with an accelerated decline in the beta cell function and to the onset of overt diabetes. Diagnostic criteria for pre-diabetes have changed over time. In the last years, the American Diabetes Association (ADA) has proposed the assessment of glycosylated hemoglobin (HbA1c) in alternative to fasting or post-load glycemia for the screening of pre-diabetes both in children and adults [4]. Consequently, pre-diabetes may be now defined by three different conditions, i.e., impaired fasting glucose (IFG), impaired glucose tolerance (IGT), and elevated levels of HbA1c (5.7–6.4%) (IA1c) or a combination of them. These phenotypes are not distinct pathological entities, but rather represent different phases of a disease that is a continuum, where each group of patients may be in a different stage of progression**.** Each phenotype, which is expression of a distinct alteration of glucose metabolism, is individually associated to a cluster of adverse cardiovascular risk factors and conveys greater risk for the development of T2DM over time. These issues are well known in adulthood but are less explored in youths.

The prevalence of pre-diabetes in children and adolescents with OB is influenced by ethnicity, age or severity of weight excess. It may also vary according to the definition adopted. A change in the overall prevalence of pre-diabetes is expected to occur by including IA1c as diagnostic criterion. Therefore, updated studies are needed to characterize the impact of the new phenotypes on the prevalence of pre-diabetes with respect to previous criteria. For instance, the National Health and Nutrition Examination Surveys (2005–2006 through 2015–2016) has recently reported updated estimates of the prevalence of pre-diabetes based on IFG, IGT, or IA1c in adolescents with OB compared to normal weight (25.7% of 16.4%, respectively) [3]. In this multi-ethnic population, a wide heterogeneity in the overall prevalence of pre-diabetes was found by race/ethnicity. In fact, a higher prevalence in Black non-Hispanic or Hispanic was reported compared to the White non-Hispanic population [3]. This finding may explain the different prevalence of pre-diabetes in the US or European children or adolescents with overweight (OW) or OB [5–11]. Therefore, country-specific studies are needed, considering that HbA1c levels may vary according to the ethnic background, lifestyle or socioeconomic conditions.

To our knowledge, no study has assessed the prevalence of pre-diabetes according to the three categories of IFG, IGT or IA1c in Italian children with OW/OB. Furthermore, few studies analyzed the association between pre-diabetes phenotypes and cardiovascular risk factors in youths [12].

Therefore, the aim of this cross-sectional multicenter study was to evaluate the prevalence of IFG, IGT and IA1c in a large sample of Italian outpatient youths with OW/OB and to compare parameters of beta cell function, insulin sensitivity and cardio-metabolic risk among these different phenotypes.

## Subjects and methods

### Participants

This retrospective multicenter study was undertaken within the Childhood Obesity study group of the Italian Society for Pediatric Endocrinology and Diabetology (ISPED). Nine tertiary Italian centers for the diagnosis and care of pediatric obesity distributed throughout the country participated and provided anthropometric and biochemical data of 1562 children and adolescents aged 5–18 years consecutively observed in the period June 2016–June 2020. Thirteen youths who showed glycemic data within the category of T2DM were excluded. Lastly, data of 1549 young people (774 boys and 775 girls) with complete anthropometric and biochemical data were analyzed. This study was approved by the Ethics Committee of the AORN Santobono-Pausilipon (reference number 22877/2020) and conformed to the guidelines of the European Convention of Human Rights and Biomedicine for Research in Children as elsewhere described. The study was also in accordance with the 1975 Declaration of Helsinki, revised in 1983, and informed consent was obtained from the parents or tutors of all participants.

### Measurement

Height and weight were measured in each center by a single trained operator as previously described [13]. Body mass index (BMI) was transformed into standard deviation score (SDS), based upon the Italian BMI percentiles [14]. Prepubertal stage was defined by Tanner Stage I of breast development in girls and testicular volume in boys [15]. After 12 h of fasting, blood samples were drawn for glucose (*G*_0_) insulin (*I*_0_) and HbA1c measurements. Oral glucose tolerance test (OGTT) was performed in the whole sample using 1.75 g/kg of glucose up to a maximum of 75 g, and two-hour post-load glucose (*G*_120_) was analyzed [4]. Data of glucose (*G*_30_) and insulin (*I*_30_) at 30′ during OGTT were available in a subsample of 952 youths. Insulin resistance (IR) was calculated by homeostatic model assessment (HOMA-IR). Insulin sensitivity (IS) was calculated as 1/*I*_0_ [16].

Insulinogenic index (IGI) was calculated as Δ(*I*_0_–*I*_30_)/Δ(*G*_0_–*G*_30_), where insulin was expressed as µU/mL and glucose as mg/dL. Disposition index (DI) was calculated according to the following formula: IGI × 1/*I*_0_ [17]

Biochemical analyses were performed in the centralized laboratory of each center. HbA1c was assessed by high-performance liquid chromatography. All laboratories belong to the Italian National Health System and are certified according to International Standards IS 000 (www.iso000.it/), undergoing to semi-annual quality controls and inter-lab comparisons.

### Definitions

Prediabetes was defined as any of the following phenotypes: impaired fasting glucose (IFG) (fasting glucose ≥ 100 < 126 mg/dL), and/or impaired glucose tolerance (IGT) (post-load glucose ≥ 140 < 200 mg/dL) and/or increased levels of glycosylated hemoglobin (IA1c) (HbA1c ≥ 5.7 < 6.5% or ≥ 39 < 48 mmol/mol), Overweight (OW) and obesity (OB) were defined on the basis of the Italian BMI standards (respectively the 75th and 95th percentiles).

IR was estimated by 97th percentile of HOMA-IR distribution by age and gender in normal weight Italian children [18]. Low IS or low DI were defined by the 25th percentile of respectively 1/*I*_0_ or DI in our sample.

Family history of T2DM was defined by the presence of T2DM in at least one among the first- or second-degree relatives.

### Statistical analysis

Continuous data were expressed as mean ± standard deviation (SD), numbers and proportions as percentage (%) and 95% confidence interval (CI). Variables with skewed distribution (i.e., HOMA-IR, 1/*I*_0_, *I*_0_, *I*_30_, IGI, DI) were log-transformed for the analysis and expressed as median and interquartile range of non-transformed values. Mean values were compared using Student’s *t* test or ANCOVA, adjusted for centers, pairwise comparisons were estimated by Sidak post hoc analysis. Distribution of categories was compared by *χ*^2^ and, when needed, exact tests were performed using the Monte Carlo method and the Bonferroni correction. The concordance between the three phenotypes was tested by Cohen’s kappa (*k*) coefficient.

The odds ratio of family history of T2DM, IR, low IS, low DI was tested using logistic regression analysis using backward procedure and age, prepubertal stage and BMI and phenotypes of pre-diabetes as covariates. A *P* value < 0.05 was considered statistically significant. The statistical analysis was performed using the IBM SPSS Statistics, Version 20.0. Armonk, NY.

## Results

The features of the whole sample and separately for boys and girls are reported in Table 1. Sex differences were found for G_0_, 1/*I*_0_ and systolic BP (higher in boys) and I_0_ and HOMA-IR (higher in girls). No differences were found for age, BMI, and BMI-SDS.

**Table 1:** Description of the sample as a whole and by gender

*n*	All	Boys	Girls	*P* value
	1549	774	775	
Age, years	11.6 ± 2.6	11.6 ± 2.5	11.6 ± 2.8	0.879
Prepubertal stage, *n* (%)	200 (13)	92 (11)	108 (14)	0.229
BMI (kg/m^2^)	30.9 ± 5.6	31.1 ± 5.5	30.7 ± 5.7	0.218
BMI-*z* score (SDS)	2.3 ± 0.6	2.3 ± 0.6	2.3 ± 0.6	0.751
Family history of T2D (%)	804 (52)	399 (52)	405 (52)	0.780
*G*_0_ (mg/dL)	88.1 ± 9.7	89.0 ± 9.4	87.3 ± 9.9	0.001
*G*_120_ (mg/dL)	110.8 ± 20.7	111.2 ± 19.2	110.5 ± 22.2	0.540
HbA1c (mmol/mol)	34.4 ± 4.3	34.4 ± 4.3	34.3 ± 4.4	0.512
HbA1c (%)	5.3 ± 0.4	5.3 ± 0.4	5.3 ± 0.4	0.512
*I*_0_ (µU/mL)	17.2 (11.9–25.1)	16.2 (11.7–23.6)	18.4 (12.0–26.4)	0.004
HOMA-IR	3.7 (2.5–5.4)	3.5 (2.4–5.1)	3.9 (2.5–5.8)	0.033
1/*I*_0_ (µU/mL)	0.06 (0.04–0.08)	0.06 (0.04–0.09)	0.05–0.04–0.08)	0.004
Cholesterol (mg/dL)	154.5 ± 29.3	154.1 ± 29.5	154.8 ± 29.1	0.618
HDL C (mg/dL)	47.0 ± 10.1	47.1 ± 10.0	46.8 ± 10.2	0.515
Triglycerides (mg/dL)	80.0 (62.0–105.0)	78.0 (61.0–104.0)	82.0 (64.0–108.0)	0.053
TG/HDL ratio	1.8 (1.3–2.4)	1.7 (1.2–2.4)	1.8 (2.3–2.5)	0.057
TC/HDL ratio	3.4 ± 0.9	3.4 ± 0.9	3.4 ± 0.9	0.303
Systolic BP (mmHg)	113.5 ± 13.9	114.8 ± 13.4	112.2 ± 14.3	< 0.0001
Diastolic BP (mmHg)	67.8 ± 9.4	68.0 ± 9.3	67.5 ± 9.6	0.281

Data are expressed as mean ± standard deviation, median (IQ range), *n* (%)

*G*_*0*_ fasting glucose, *G*_*120*_ glucose at 120′ during OGTT, *I*_*0*_ fasting insulin, *HOMA-IR* homeostasis model assessment, *1/I*_*0*_ reciprocal of fasting insulin, *TG/HDL* ratio triglycerides-to-HDL ratio, *TC/HDL* ratio total cholesterol-to-HDL ratio

**Fig. 1:**
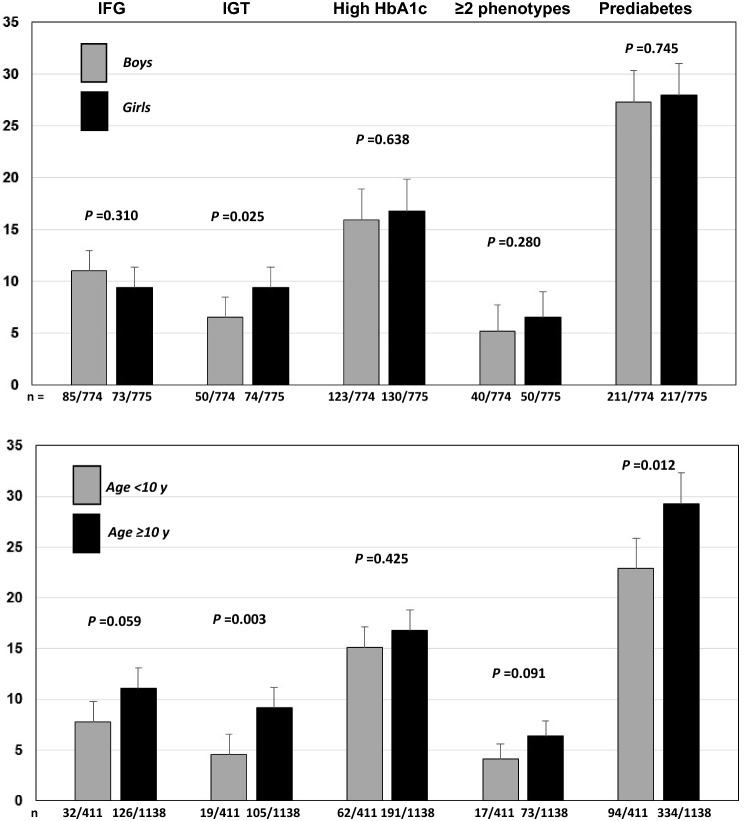
Prevalence of pre-diabetes and its phenotypes by gender (top panel) and by age (bottom panel)

The percentages of isolated or any combined presence of two or more phenotypes are reported in Table 2. The most frequent phenotype was represented by IA1c, either isolated or combined with other phenotypes. The percentage of isolated IFG and IGT was quite similar, while IFG was slightly more frequent than IGT, when it was associated with any other phenotype. Prevalence of pre-diabetes and its phenotypes by gender and by age groups is shown in Fig. 1. Regarding gender distribution, a higher prevalence of IGT was observed in girls vs boys (*P* = 0.025). Regarding age distribution, a higher prevalence of IGT and pre-diabetes was observed in adolescents (age ≥ 10 years) vs children (age < 10 years). A similar trend (albeit not statistically significant) was observed for IFG (*P* = 0.059). On the contrary, no age-related difference was observed for IA1c.

**Table 2:** Prevalence of phenotypes of pre-diabetes

	*n*	% (Cl)
Normoglycemic individuals	1121	72.4 (70.1–74.6)
IFG	158	10.2 (8.7–11.7)
IGT	124	8.0 (6.7–9.4)
IA1c	253	16.3 (14.5–18.2)
Isolated IFG	83	5.4 (4.2–6.5)
Isolated IGT	78	5.0 (4.0–6.1)
Isolated IA1c	177	11.4 (9.8–13.1)
Two or more phenotypes	90	5.8 (4.7–7.0)
IFG + IGT	14	0.9 (0.4–1.4)
IFG + IA1c	44	2.8 (2.0–3.7)
IGT + IA1c	15	1.0 (0.5–1.5)
IFG + IGT + IA1c	17	1.1 (0.6–1.6)
Any pre-diabetic phenotype	428	27.6 (25.4–29.9)

Data are expressed as numbers (95% Cl)

The agreement between phenotypes was poor (*k* index 0.14 between IFG and IGT, 0.20 between IFG and IA1c, and 0.07 between IGT and IA1c).

The features of whole sample divided according to the absence of any glucose derangements, the isolated presence or any combined presence of IFG, IGT and IA1c are reported in Table 3. As expected, *G*_0_, *G*_120_, and HbA1c differed among groups by definition. Furthermore, groups differed significantly for age, family history of T2DM, and BMI, but not for BMI-SDS. The group with ≥ 2 phenotypes exhibited the oldest age, the highest BMI-SDS, *I*_0_ and HOMA-IR and the lowest insulin sensitivity (1/*I*_0_) compared to the other groups. On the contrary, the group without pre-diabetes showed a better cardio-metabolic profile, with lower levels of both lipids and BP.

**Table 3:** Anthropometric, clinical, and biochemical variables among phenotypes of pre-diabetes

*n* = 1549	No pre-diabetes (*n* = 1121)	Isolated IFG (*n* = 83)	Isolated IGT (*n* = 78)	Isolated IA1c (*n* = 177)	≥ 2 phenotypes (*n* = 90)
Male gender, *n* (%)	563 (50)	52 (63)^a^	32 (41)	87 (49)	40 (44)
Prepubertal stage, *n* (%)	162 (14)	10 (12)	4 (5)^a^	17 (10)^a^	7 (8)
Family history, *n* (%)	586 (52)	44 (53)	54 (69)^b^	77 (44)	43 (48)
Age (years)	11.5 ± 2.7	11.8 ± 2.6	12.2 ± 2.4	11.5 ± 2.5	12.4 ± 2.6
BMI, kg/m^2^	30.7 ± 5.4	31.7 ± 6.0	31.0 ± 5.3	31.2 ± 6.1	32.4 ± 6.2^b^
BMI-SDS	2.3 ± 0.6	2.4 ± 0.6	2.3 ± 0.6	2.3 ± 0.6	2.5 ± 0.6^b^
*G*_0_, (mg/dL)	85.8 ± 8.1	103.9 ± 4.0^c^	89.0 ± 7.2^c^	87.5 ± 7.2	102.7 ± 8.8^c^
*G*_120_, (mg/dL)	105.2 ± 15.6	117.3 ± 13.1^c^	153.3 ± 13.2^c^	109.8 ± 16.2^c^	139.8 ± 26.7^c^
HbA1c (mmol/mol)	33.0 ± 3.7	34.3 ± 2.9^b^	33.2 ± 3.4	40.3 ± 1.6^c^	40.1 ± 2.7^c^
HbA1c (%)	5.2 ± 0.3	5.3 ± 0.3^b^	5.2 ± 0.3	5.8 ± 0.1^c^	5.8 ± 0.2^c^
*I*_0_ (µUI/mL)	16.1 (11.4–23.7)	17.9 (12.5–25.9)	20.4 (13.7–34.1)^c^	19.3 (12.8–26.6)	24.8 (14.6–36.3)^c^
HOMA-IR	3.4 (2.3–4.9)	4.5 (3.1–6.8)^c^	4.5 (3.1–7.3)^c^	4.1 (2.8–5.9)	6.2 (3.8–9.0)^c^
1*/I*_*0*_ (µUI/mL)	0.06 (0.04–0.09)	0.06 (0.04–0.08)	0.05 (0.03–0.07)^c^	0.05 (0.04–0.08)	0.04 (0.03–0.07)^c^
Cholesterol, (mg/dL)	154.1 ± 29.4	154.1 ± 33.5	154.8 ± 30.4	156.3 ± 27.9	155.1 ± 25.5
HDL C (mg/dL)	48.1 ± 10.5	44.2 ± 7.9^c^	43.2 ± 7.3^c^	44.3 ± 7.4^c^	44.3 ± 10.3^c^
Triglycerides (mg/dL)	78.0 (61.0–102.0)	86.0 (60.0–108.0)	87.0 (66.0–115.0)	89.0 (69.0–112.5)^b^	88.0 (67.0–111.3)
TG/HDL ratio	1.7 (1.2–2.3)	2.0 (1.3–2.6)	2.0 (1.5–2.7) ^c^	2.0 (1.4–2.8)^c^	2.0 (1.5–2.7)^c^
TC/HDL ratio	3.3 ± 0.9	3.6 ± 0.8	3.7 ± 1.0^c^	3.6 ± 0.8^c^	3.6 ± 0.9^c^
Systolic BP (mmHg)	113.1 ± 13.8	114.6 ± 12.9	115.6 ± 14.8	112.4 ± 14.8	117.7 ± 12.7
Diastolic BP (mmHg)	67.6 ± 9.4	69.1 ± 9.5	66.6 ± 9.3	66.7 ± 9.3	71.8 ± 9.8

Data are expressed as mean ± standard deviation, median (IQ range), *n* (%)

^a^*P* < 0.05 vs no pre-diabetes

^b^*P* < 0.025 vs no pre-diabetes

^c^*P* < 0.001 vs no pre-diabetes

In the subsample of 958 youths in whom the IGI was available, the isolated IGT phenotype was characterized by more prevalent relatives with T2DM, and lower values of IGI and DI (Table, Supplementary materials).

The proportions of youths with IR, low IS and low DI, across the different pre-diabetes phenotypes are reported in Fig. 2 of Supplementary materials.

The strength of the associations (expressed as Odds Ratios adjusted for centers, age, prepubertal status, and BMI) between phenotypes of pre-diabetes and metabolic abnormalities or markers of atherogenic dyslipidemia is shown in Table 4. Compared to youths without pre-diabetes (reference category), family history of T2DM was associated only with isolated IGT, while the abnormalities underlying beta cell function and insulin sensitivity were associated with either isolated IGT or any combined phenotype. On the contrary, only IR was associated with isolated IFG, while IR and low DI were associated with IA1c, but at a lesser extent than isolated IGT.

**Table 4:** Odds Ratio of family history of type 2 diabetes mellitus, insulin resistance, low insulin sensitivity and low oral disposition index associated with phenotypes of pre-diabetes

	No pre-diabetes	Isolated IFG	Isolated IGT	Isolated IA1c	≥ 2 phenotypes
Family history*	1.00	1.17 (0.70–1.94)	2.06 (1.20–3.55)^c^	1.05 (0.74–1.49)	1.27 (0.79–2.05)
High HOMA-IR*	1.00	2.17 (1.23–3.82)^c^	2.63 (1.47–4.70)^c^	1.64 (1.12–2.40)^c^	3.91 (2.04–7.49)^c^
Low IS*	1.00	1.10 (0.64–1.88)	2.54 (1.56–4.15)^c^	1.31 (0.90–1.93)	2.91 (1.81–4.68)^c^
Low DI**	1.00	1.18 (0.65–2.11)	3.21 (1.83–5.64)^c^	1.73 (1.04–2.90)^a^	2.37 (1.38–4.07)^c^
High TG/HDL ratio	1.00	1.31 (0.80–2.14)	1.94 (1.19–3.16)^c^	2.05 (1.44–2.94)^c^	1.20 (0.74–1.96)
High TC/HDL ratio	1.00	1.22 (0.73–2.04)	1.91 (1.16–3.16)^b^	1.74 (1.20–2.54)^c^	1.40 (0.85–2.28)

^*^P value adjusted for centers, age, prepubertal stage and BMI, **P value adjusted for center

^a^*P* < 0.05 vs no pre-diabetes

^b^*P* < 0.025 vs no pre-diabetes

^c^*P* < 0.001 vs no pre-diabetes

## Discussion

The present study has demonstrated that more than a quarter of Italian children and adolescents with OW/OB had pre-diabetes and that IA1c was the most prevalent phenotype. Compared to the other phenotypes, isolated IGT was strongly associated with family history of T2DM and showed the worst metabolic profile in terms of insulin resistance, low insulin sensitivity, low DI and atherogenic lipid profile.

The assessment of A1c for the diagnosis of pre-diabetes is still supported by limited data in children, as underlined by the most recent ADA guidelines [4]. However, there are many advantages of using A1c, since it does not require fasting, has low intra-individual variability, and it is a good predictor of diabetes-related complications in adults [19]. Several studies have assessed the prevalence of IA1c in the pre-diabetes range and the potentiality of this biochemical marker to predict T2DM in youths with obesity compared to FPG and 2hPG, yielding contrasting results [20–23].

In our sample, the overall prevalence of IA1c was 16.3%, representing the most frequent phenotype and accounting for 50% of the isolated phenotypes. Compared to our study, a higher prevalence of IA1c (21% and 23.6% respectively) was reported in two clinical studies, the former conducted in a multi-ethnic cohort of 1156 US youths with obesity [20], the latter in a clinical sample of 4848 children and adolescents with OW and OB [23]. On the contrary, a lower prevalence of IA1c (12.2%) was reported in OW/OB youths from the NANHES 2005–2016 population study [3]; in this group, IFG accounted for 29.2% and IGT for 9.0%. Discrepancies among studies are difficult to explain, since the threshold used to define IA1c was the same (5.7–6.4%) and the age range was quite similar. Probably, different settings (population versus clinical samples) and the presence of multi-ethnic populations might account for the different results.

We observed 5.4% cases with isolated IFG, which is by far lower than 26% reported in the NANHES 2005–2016 [3]. This finding is not surprising since the prevalence of isolated IFG among obese young people is widely heterogeneous among studies, ranging between 0.4% in youths of European origin [24] and 35.8% in a Swedish cohort [10]. On the contrary, the prevalence of isolated IGT was only slightly lower (5% vs 8.1%) than that reported by the NANHES 2005–2016 [3]. Indeed, the prevalence of IGT is much more consistent across different studies, ranging from 3.2% in a Sardinian population [8] to 14.2% in the Swedish cohort [10]. Of note, our study showed that the prevalence of IGT was doubled compared to what reported more than 20 years ago by Invitti et al. in Italian children and adolescents with severe obesity [26], supporting that the prevalence of pre-diabetes is rising in Italy with the increasing prevalence of obesity.

Gender differences in the prevalence of the pre-diabetes phenotypes have been reported in adults [24, 25], while few data are available in the young population with OW/OB. In agreement with the studies performed in adults, we found that girls were more likely to have isolated IGT than boys and that no gender difference occurred with regard to IA1c. Other studies performed in children with OB reported similar findings for both IGT (in children but not in adolescents) [10]. Indeed, we did not confirm the higher male prevalence for IFG as reported in adults, but in this regard, equivocal results have been described in the pediatric literature [26–28].

Regarding the influence of age, very few studies have assessed the prevalence of pre-diabetes in children under 10 years of age [1, 28, 29]. This is not surprising, since the ADA recommended the screening of diabetes after the age of 10 years or in the presence of pubertal signs. We observed that pre-diabetes was already present in 22.8% children, although at a significantly lower extent than adolescents. This finding was true for IFG (*P* = 0.059) and especially for IGT (*P* = 0.003). Similar to our findings, Hagman et al. [29] showed an age-dependent increase of IFG risk in two nationwide cohorts (German and Swedish) of children and adolescents with OB.

Although pre-diabetes can be transient specifically at such a young age, our findings suggest the utility of pre-diabetes screening in children with OW/OB less than 10 years as an opportunity of increasing parental engagement in weight control [30].

Several studies have demonstrated a higher prevalence of family history for T2DM in youths with OB and pre-diabetes, without differentiating among phenotypes [6, 31]. We found that 52% of our sample had family history for T2DM. Indeed, this is one of the risk factors for diabetes included in the ADA criteria for screening. Interestingly, the IGT group had a significantly higher positivity of relatives with T2DM compared to the other phenotypes. Similarly, Poon et al. reported a family history of diabetes in 80% of youths with pre-diabetes (IFG and/or IGT) as compared to 53% of normoglycemic youths in a Chinese population with OW/OB [32]. With specific regard to IGT, Goran et al. reported that 28% of overweight Latino children with a positive family history of T2DM already have IGT [7]. The strong association between family history of T2DM and IGT suggests that genetic background has greater penetrance in youths with IGT than young people with the other phenotypes.

We observed a poor agreement between IFG, IGT and IA1c, as previously demonstrated in young people with OW/OB [33, 34]. This finding indicates that the three phenotypes may reflect distinct features of glucose metabolism and different stages of the pathophysiological mechanisms [35]. Indeed, IFG was associated only with IR, whereas IGT was exhibited IR, low IS, and DI. Similar characteristics were observed in individuals with ≥ 2 phenotypes.

Interestingly, the IA1c phenotype showed an intermediate risk profile compared to the other phenotypes. Hence, we can hypothesize that IA1c may detect a population at an earlier stage risk to develop T2DM compared to IGT. Since the reversal from pre-diabetes to normoglycemia is common in adolescents, especially in those with IFG, A1c is likely to be biomarker more reliable then IFG over time. Indeed, a lower regression to normoglycemia was reported in adults with IA1c compared to the other phenotypes [36], supporting the hypothesis that this phenomenon may be dependent on a greater stability of IA1c. If this evolution is also confirmed by prospective studies in childhood, the utility of HbA1c monitoring could be crucial in adolescents at high risk of developing diabetes. Prediabetes in adolescents has been associated with a worse cardiovascular profile [3, 37]. Interestingly, by analyzing the single phenotypes, we found a significant association between isolated IGT or IA1c and biomarkers of atherogenic risk. The present study extends our previous findings on the worse cardiovascular profile in obese youths with IGT [11] to include also the IA1c phenotype, highlighting that both phenotypes represent categories metabolically distinct from IFG.

Our study presents some limitations. The cross-sectional design does not allow inferences on the progression of each phenotype of pre-diabetes, in addition the prevalence of pre-diabetes is limited to categories of youths with OW/OB and cannot be extended to the general population. Furthermore, HbA1c and insulin measurements were not centralized in a single laboratory, thus variability between the different centers could not be totally excluded. In addition, our data were obtained in a sample of Caucasian youths, therefore they cannot be easily compared to other studies based on multi-ethnic populations. However, this feature may represent also a strength of our study, since the prevalence of pre-diabetes in Caucasian populations is by far less explored. Furthermore, the comprehensive analysis of metabolic characteristics of each phenotype and their association with the atherogenic major cardio-metabolic risk factors may represent another strength of our study.

## Conclusion

Our study has demonstrated that in an outpatient population of youths with OW/OB the prevalence of pre-diabetes is high and that IA1c was the most prevalent phenotype. IA1c showed intermediate characteristics in terms of both metabolic impairment and atherogenic risk profile as compared to IFG and IGT. More studies are needed to assess whether A1c may help improving the prediction of diabetes.

## Supplementary Information

Below is the link to the electronic supplementary material.

Supplementary file1 (DOCX 143 KB)

## Data Availability

De-identified individual participant data will not be made available.

## Abbreviations

1/*I*_0_: Reciprocal of fasting insulin
ADA: American Diabetes Association
BMI: Body mass index
CMR: Cardiometabolic risk
DBP: Diastolic blood pressure
*G*_0_: Fasting glucose
*G*_120L_: Glucose at 120′ during OGTT
HbA1c: Glycosylated hemoglobin
HOMA-IR: Homeostasis model assessment of insulin resistance
*I*_0_: Fasting insulin
IA1c: Increased HbA1c
IFG: Impaired fasting glucose
IGT: Impaired glucose tolerance
ISPED: Italian Society of Pediatric Endocrinology and Diabetology
OB: Obesity
OGTT: Oral glucose tolerance test
OR: Odds ratio
OW: Overweight
SBP: Systolic blood pressure
TC/HDL ratio: Total cholesterol-to-HDL ratio
