# Supplementary material for: Phenotypes of prediabetes and metabolic risk in Caucasian youths with overweight or obesity
Source: J Endocrinol Invest. 2022 May 17;45(9):1719–27. doi: 10.1007/s40618-022-01809-3 (PMC9360115; doi:10.1007/s40618-022-01809-3)
Supplement: Supplementary file 1 — Supplementary file1 (DOCX 143 KB) [file 40618_2022_1809_MOESM1_ESM.docx]

**Supplemental table**. Anthropometric, clinical, and biochemical variables among phenotypes of prediabetes in a subsample of youths with evaluation at 30’ during OGTT

|  | **No prediabetes** | **Isolated IFG** | **Isolated IGT** | **Isolated IA1c** | **≥2 phenotypes** | ***P* value** |
| --- | --- | --- | --- | --- | --- | --- |
| ***n =958*** | ***657*** | ***72*** | ***57*** | ***100*** | ***72*** |  |
| Male gender , n (%) | 335 (51) | 47 (65) | 27 (47) | 49 (49) | 30 (42) | 0.065 |
| Prepubertal stage, n (%) | 78 (12) | 7 (10) | 4 (7) | 9 (9) | 4 (6) | 0.388 |
| Family history, n (%) | 388 (59) | 37 (51) | 40 (70) | 39 (39) | 32 (44) | <0.0001 |
| Age (years) | 11.8±2.6 | 11.9±2.5 | 12.3±2.6 | 12.0±2.5 | 12.4±2.4 | 0.178 |
| BMI, Kg/m^2^ | 30.8±5.5 | 31.7±6.0 | 30.8±5.5 | 30.6±5.0 | 32.4±6.5 | 0.121 |
| BMI-SDS | 2.3±0.6 | 2.4±0.7 | 2.3±0.6 | 2.3±0.6 | 2.5±0.6 | 0.179 |
| G_30_ (mg/dL) | 131.1±19.3 | 145.2±18.3 | 145.0±22.4 | 134.1±21.4 | 150.1±21.7 | <0.0001 |
| I_30_ (µUI/ml) | 113.0 (70.0-180.0) | 110.4 (68.0-191.7) | 121.2 (84.8-190.5) | 126.4 (79.9-184.8) | 137.3 (78.0-210.1) | 0.256 |
| IGI | 2.5 (1.5-4.0) | 2.5 (1.4-4.5) | 2.2 (1.2-3.3) | 2.4 (1.7-3.9) | 2.4 (1.6-4.0) | 0.250 |
| DI | 0.15 (0.10-0.23) | 0.14 (0.09-0.22) | 0.10 (0.07-0.16) | 0.13 (0.08-0.19) | 0.11 (0.07-0.17) | <0.0001 |

Data are expressed as mean±standard deviation, median (IQ range), n (%).

**Supplemental figure.** Proportion of individuals with insulin resistance, low insulin sensitivity and low disposition index among categories of prediabetes: no prediabetes (white bars), IFG (grey bars), IGT (chess bars), IA1c (dark chess bars), ≥2 phenotypes (black bars)

**Low disposition index**

**Low insulin sensitivity**

**Insulin resistance**

**
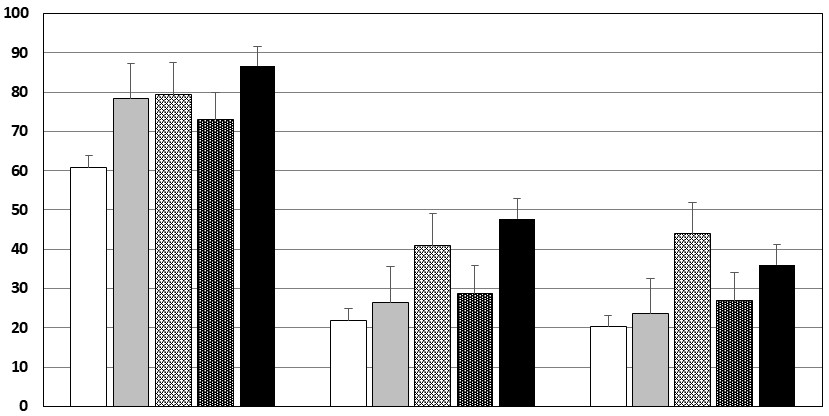
**

***P* <0.0001**

***P* <0.0001**

***P* <0.0001**

**n = 683/1121 65/83 62/78 129/177 78/90 246/1121 22/83 32/78 51/177 43/90 133/657 17/72 25/57 27/100 26/72**
